# Seroprevalences of multi-pathogen and description of farm movement in pigs in two provinces in Vietnam

**DOI:** 10.1186/s12917-020-2236-7

**Published:** 2020-01-14

**Authors:** Hu Suk Lee, Vuong Nghia Bui, Huyen Xuan Nguyen, Anh Ngoc Bui, Trung Duc Hoang, Hung Nguyen-Viet, Delia Grace Randolph, Barbara Wieland

**Affiliations:** 1 International Livestock Research Institute (ILRI), Room 301-302, B1 Building, Van Phuc Diplomatic Compound, 298 Kim Ma Street, Ba Dinh District, Hanoi, Vietnam; 2 National Institute of Veterinary Research, 86 Truong Chinh, Phuong Mai, Dong Da, Hanoi, Vietnam; 3 International Livestock Research Institute (ILRI), Nairobi, Kenya; 4 International Livestock Research Institute (ILRI), Addis Ababa, Ethiopia

**Keywords:** Vietnam, Pigs, Sero-prevalence, Co-infection, Farm movement

## Abstract

**Background:**

In Vietnam, lack of animal health information is considered a major challenge for pig production. The main objective of this study was to assess the seroprevalences of five pathogens [porcine circovirus type 2 (PCV2), porcine reproductive and respiratory syndrome virus (PRRSV), mycoplasma hyopneumoniae (M. hyo), Japanese encephalitis virus (JEV) and leptospirosis] and to better characterize the farm movements through a survey.

**Results:**

A total of 600 samples were collected from 120 farms from Bac Giang and Nghe An. Among unvaccinated herds, the highest seroprevalence was found for JE with 73.81% (95% CI: 68.39–78.74) in Bac Giang and 53.51% (95% CI 47.68–59.27) in Nghe An. Seroprevalences for PCV2 and M.hyo were 49.43% (95% CI: 45.06–53.80) and 46.06% (95% CI: 41.48–50.69) among unvaccinated animals. Accumulative co-infections for JE (86.25%) showed the highest level followed by M. hyo (66.25%) and PCV2 (62.50%). Three co-infections with JE had the highest positive rate (28.75%) followed by four co-infections (25.0%). Medium farms had relatively higher herd prevalences for all pathogens, except from leptospirosis. Overall, farmers exported/imported their pigs at the most 1–2 times every 6 months. Some respondents (5% for exportation and 20% for importation) had moved pigs more than 6 times over the last 6 months.

**Conclusions:**

Our study provided another pool of evidence that showed that PCV2, PRRS and H. hyo are endemic in pigs in Vietnam. Given the economic impacts of these pathogens elsewhere, the findings confirm the need for studies to evaluate the association between antibody response and clinical relevance as well as to assess the economic impact of co-infections at farm level. We also found that high seroprevalences of JE and leptospirosis were detected in pigs. From a pubic health point of view, it is crucial to raise public awareness especially for high risk occupations (mainly pig farm workers).

## Background

In Vietnam, several constraints to pig production have been identified, most importantly animal diseases, lack of veterinary services, poor nutrition and inadequate animal productivity/genetic make-up [1]. Poor access to animal health information is considered a major challenge for pig production. In addition, more than 70% of producers are smallholders and have low awareness and knowledge on potential disease transmission pathways and biosecurity.

Vietnam’s national animal health surveillance program consists primarily of local authorities who collect data daily/weekly (through email, fax and hard copy paper forms) on major animal production diseases only. These include, classical swine fever (CSF), Foot-and-mouth (FMD) disease and porcine reproductive and respiratory syndrome (PRRS). Most cases are reported from small and medium holder farms via passive surveillance. Among reported cases, only a few are confirmed by laboratories while most reported cases are clinically diagnosed by local animal health workers due to lack of diagnostic facilities in rural areas. It can be assumed therefore that most diseases are underreported under the current national surveillance program.

A few epidemiological and serological surveys have been conducted for major production diseases in pigs in Vietnam [2–5]. Recently, using national animal health data, the first national PRRS study was conducted to identify seasonal patterns and clusters [6]. In the area of zoonotic diseases, while leptospirosis is a notifiable disease in humans, only few cases have been reported to the national surveillance system in Vietnam even though the disease is considered to be endemic [7–9]. A recent study showed that the sero-prevalence was 21.05% in pigs from 10 provinces [8]. Japanese encephalitis (JE) is a vector-borne zoonotic disease and pigs are considered to play an important role in amplifying hosts for transmission to humans given that pigs are often raised near human habitations [10]. A recent study showed that the sero-prevalence of JE in pigs was 73.45% [8].

Previous studies suggest that the major risk factors for animal diseases are the regular movement of animals between farms [11–14]. Moreover, transportation and fomites (e.g. body fluids, soil and droplet) can play a role in disease transmission [15–17]. Therefore, it is very important to understand the movement patterns at farm level.

To our knowledge, few studies in Vietnam have looked at a range of pathogens simultaneously. Therefore, the main objective of this study was to assess the seroprevalences of five pathogens and better characterize the farm movements through a survey.

## Results

Farm survey and description of farm movement.

A total of 120 pig farmers [26 (Female): 94 (Male)] were interviewed from two provinces (60 farmers/province) (Additional file 1). The median age of respondents was 49.5 and the range was 25–90 years old. Almost 90% of people reached either primary to high school education levels. A total of 74.17% farms were classified into small farms whereas large farms accounted for 4.17%. Overall, farmers exported/imported their pigs at most 1–2 times every 6 months (Table 1). Some respondents (5% for exportation and 20% for importation) had moved pigs more than 6 times over the last 6 months. More than 60% of pig farms use the continuous flow systems. Veterinarians and animal health workers visited farms more than 2 twice every 6 months while most of farmers (88%) did not allow vehicles onto their premises.

**Table 1:** Summary of farm movement information through survey

Questions	Category	Proportion (%)
How often did you import the pigs on your farm over the last 6 months?	0	41.67
	≤2 times	41.67
	> 2 times	16.67
How often did you export your pigs from your farm over the last 6 months?	0	9.17
	≤2 times	53.33
	> 2 times	37.5
What kind of production system do use on your farm?	All-in-All-out	35.83
	Continuous flow	64.17
Are new purchased pigs that are mixed with existing pigs over the last 6 months?	Yes	15.83
	No	84.17
How often did other pig farmers visit on your farm over the last 6 months?	0	81.67
	≤2 times	7.50
	> 2 times	10.83
How often did traders visit on your farm over the last 6 months	0	25.0
	≤2 times	35.83
	> 2 times	39.17
How often did butchers visit on your farm over the last 6 months?	0	63.33
	≤2 times	20.0
	> 2 times	16.67
How often did veterinarians/animal health workers visit on your farm over the last 6 months?	0	40.83
	≤2 times	40.83
	> 2 times	18.33
How often were vehicles allowed onto the premises over the last 6 months?	0	88.33
	≤2 times	7.50
	> 2 times	4.17

### Seroprevalences of multi-pathogen

**Fig. 1:**
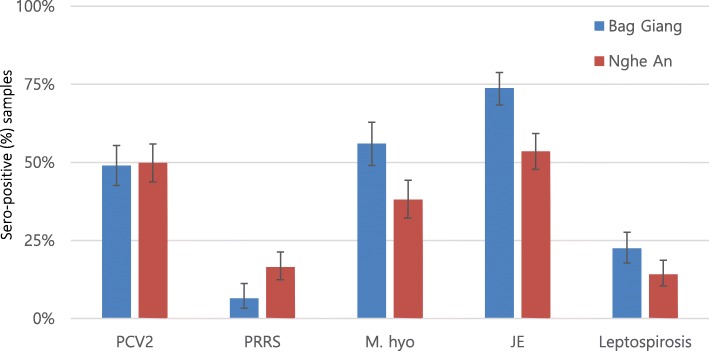
Sero-prevalences with 95% CI of multi-pathogen in unvaccinated pigs in two provinces

A total of 600 samples were collected from 120 farms across the two provinces. Six samples from Bac Giang (three samples each farm from two farms) and one sample from Nghe An could not be analyzed due to hemolysis, while sufficient sera for 9 samples were not available to perform the microscopic agglutination test (MAT) for leptospirosis. The highest seroprevalence was found for JE with 73.81% [95% confidence interval (CI): 68.39–78.74] in Bac Giang and 53.51% (95% CI 47.68–59.27) in Nghe An (to note: none of the herds were vaccinated against JE). Seroprevalences for porcine circovirus type 2 (PCV2) and mycoplasma hyopneumoniae (M. hyo) were 49.43% (95% CI: 45.06–53.80) and 46.06% (95% CI: 41.48–50.69), respectively whereas leptospirosis and PRRS showed the lowest seroprevalences among unvaccinated animals (Fig. 1).

**Fig. 2:**
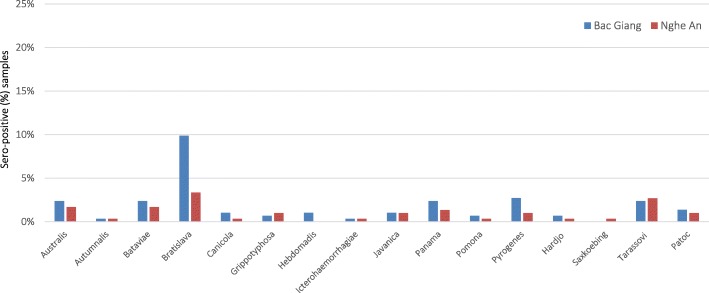
Sero-positive samples by *Leptospira* serovar in two provinces using cut off titer ≥1:100

Vaccinated herds with PRRS showed the highest seropositive rate (22.5%) followed by M. hyo (20.83%) and PCV2 (11.67%) whereas none of the herds were vaccinated with JE and leptospirosis (Table 2). Seropositive rates between vaccinated and unvaccinated herds were 55.56% (15 of 27):17.20% (16 of 93) for PRRS and 88.0% (22 of 25): 69.47% (66 of 95) for M. hyo. Overall, medium farms had relatively higher herd prevalences for all pathogens, except from leptospirosis. All medium farms were infected with M. hyo and JE. For leptospirosis, the most frequently detected infective serovar was Bratislava (6.60%), followed by Tarassovi (2.54%), Australis (2.03%) and Bataviae (2.03%) using a cut-off titer of ≥1:100 (Table 3). In Bac Giang, serovar Bratislava had the highest prevalence (9.86%) followed by Pyrogenes (2.72%) while Bratislava (3.37%) had the highest followed by Tarassovi (2.69%) in Nghe An (Fig. 2).

**Table 2:** Herd prevalence by farm type [small (< 100), medium (< 500) and large (≥ 500)] in vaccine and unvaccinated farms

Tested pathogen	No. of vaccinated farm type	Seroprevalence of vaccinated farms with 95% CI	No. of unvaccinated farm type	Seroprevalence of unvaccinated farms with 95% CI
PCV2	Small (6)	50.0 (11.81–88.19)	Small (83)	57.83 (46.49–68.60)
	Medium (6)	100.0 (54.07–100.0)	Medium (20)	80.0 (56.34–94.27)
	Large (2)	100.0 (15.81–100.0)	Large (3)	33.33 (0.84–90.57)
PRRS	Small (12)	41.67 (15.17–72.33)	Small (77)	9.09 (3.73–17.84)
	Medium (13)	69.23 (38.57–90.91)	Medium (13)	61.54 (31.58–86.14)
	Large (2)	50.0 (1.26–98.74)	Large (3)	33.33 (0.84–90.57)
M. hyo	Small (14)	78.57 (49.20–95.34)	Small (75)	64.0 (52.09–74.77)
	Medium (9)	100.0 (66.37–100.0)	Medium (17)	100.0 (80.49–100)
	Large (2)	100.0 (15.81–100.0)	Large (3)	33.33 (0.84–90.57)
JE	Small (0)	0	Small (89)	87.64 (78.96–93.67)
	Medium (0)	0	Medium (26)	100.0 (86.77–100)
	Large (0)	0	Large (5)	100.0 (47.81–100)
	Small (0)	0	Small (88)	54.55 (43.58–65.20)
Leptospirosis^a^	Medium (0)	0	Medium (25)	64 (42.52–82.03)
	Large (0)	0	Large (5)	80.0 (28.36–99.49)

^a^sera samples from two farms were not enough volumes for the MAT

**Table 3:** MAT results for Leptospria serovars in pigs using 2 cutoff titers

Serovar	Total samples	≥ 1:100	≥ 1:200
		N (%, 95 CI)	N (%, 95 CI)
Australis	591	12 (2.03, 1.05–3.52)	0
Autumnalis	591	2 (0.3, 0.04–1.21)	1 (0.17, 0.004–0.94)
Bataviae	591	12 (2.03, 1.05–3.52)	2 (0.3, 0.04–1.21)
Bratislava	591	39 (6.60, 4.73–8.91)	3 (0.5, 0.1–1.48)
Canicola	591	4 (0.68, 0.18–1.72)	0
Grippotyphosa	591	5 (0.8, 0.28–1.96)	0
Hebdomadis	591	3 (0.5, 0.1–1.48)	0
Icterohaemorrhagiae	591	2 (0.3, 0.04–1.21)	0
Javanica	591	6 (1.02, 0.37–2.20)	1 (0.17, 0.004–0.94)
Panama	591	11 (1.86, 0.93–3.31)	1 (0.17, 0.004–0.94)
Pomona	591	3 (0.5, 0.1–1.48)	0
Pyrogenes	591	11 (1.86, 0.93–3.31)	0
Hardjo	591	3 (0.5, 0.1–1.48)	0
Sakoebing	591	1 (0.17, 0.004–0.94)	0
Tarassovi	591	15 (2.54, 1.42–4.15)	1 (0.17, 0.004–0.94)
Patoc	591	7 (1.18, 0.48–2.43)	2 (0.3, 0.04–1.21)

### Co-infections

**Fig. 3:**
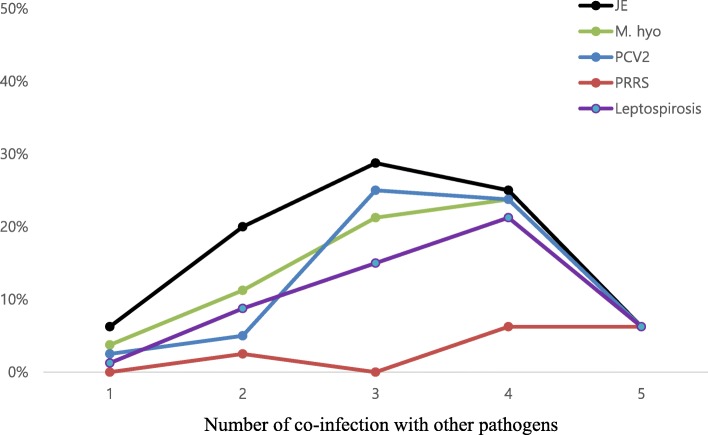
Accumulative co-infection trends of five tested pathogens among unvaccinated herds from two provinces

Table 4 demonstrated the proportion of co-infection with different pathogens among unvaccinated farms. The most common co-infections were PRRS-JE (positive rate:16/16, 100%) and JE-leptospirosis (positive rate: 64/68, 94.12%) whereas the least common co-infections were PCV2-PRRS (positive rate: 9/65, 13.84%) and M. hyo-PRRS (positive rate: 9/66, 13.64%). Overall, accumulative co-infections for JE (86.25%) showed the highest level followed by M. hyo (66.25%) and PCV2 (62.50%) (Fig. 3). Three co-infections with JE had the highest positive rate (28.75%), followed by four co-infections (25.0%). A total of five farms (medium farms: 4 and small farms:1) were infected with five pathogens, accounting for 6.25% among unvaccinated farms.

**Table 4:** Proportion of co-infection by each positive pathogen among unvaccinated farms in two provinces

Pathogen (No. of positive samples)	PCV2	PRRS	M. hyo	JE	Leptospirosis
PCV2 (65)	N/A	9 (13.84%)	41 (63.08%)	60 (92.31%)	40 (61.54%)
PRRS (16)	9 (56.25%)	N/A	9 (56.25%)	16 (100.0%)	10 (62.5)
M. hyo (66)	41 (62.12%)	9 (13.64%)	N/A	60 (90.91%)	35 (53.03%)
JE (109)	60 (55.05%)	16 (14.68%)	60 (55.05%)	N/A	64 (58.72%)
Leptospirosis (68)	40 (58.82%)	16 (23.53%)	35 (51.47%)	64 (94.12%)	N/A

## Discussion

Our study found PCV2 (50%) and M. hyo (35%) detection rates among unvaccinated pigs, similar to a previous study, conducted in Hoa Binh and Vinh Phuc whereas our study showed a relatively lower seroprevalence of PRRS (21%) [18]. Another study in southern Vietnam found a prevalence of 25% for PRRS in young pigs, age 4–5 months [19]. PCV2 is characterized by wasting, pale skin, respiratory distress and diarrhoea [20, 21]. Also, PCV2 is associated with the porcine respiratory disease complex (PRDC) [22], underlying its relevance for productivity at farm level. Similarly to Kim et al., we found that co-infection of PCV2 and M. hyo was common. In Vietnam, a study was conducted to evaluate the molecular characterization of PCV2, showing multi circulating clusters of 1A, 1B, 1C and a recombinant cluster [5].

Our study found a high seroprevalence for M. hyo infection and the likelihood that this pathogen is a cause of respiratory disease in pigs in Vietnam, which has been underestimated and for which awareness is likely to be low. It can be differentiated from PRRS by number of infected pigs affected in herd, clinical signs (e.g. local pneumonia) and spread patterns [23]. The first clinical signs of infected pig herds are coughing, anorexia and shortness of breath. Also, one study reported that the disease spreads slowly within herds, showing 20% of morbidity and 12% of fatality in Vietnam [24].

In 2006, a new strain of PRRS (called “highly pathogenic (HP) PRRS) was detected in China for the first time, characterized by high morbidity and mortality rates [25]. In 2007, HP-PRRS virus was first identified in Hai Dung province, which then spread to other provinces in Vietnam [26]. In 2008, more than 300,000 pig deaths (including controlled pig culling) were recorded in 26 out of 62 provinces which had a huge economic impact on production [27]. Since then, more pig farmers have opted to vaccinate their herds against PRRS vaccination. However, vaccinations are more commonly used in large pig farms, but not in the small and medium farms, that account for more than 95% of total pig production – and are able to sell pigs without PRRS vaccination certification. Our study showed that only 22% of small/medium farms were vaccinating their pig herds against PRRS, compared to 67% in large farms.

From a public health point of view, it has been suggested that infection of PRRS in pigs was increased by co-infection with *Streptococcus suis* (*S. suis*) [28, 29], further to one experiment that showed that PRRS infected pigs were more susceptible to *S. suis* [30]*.* In Vietnam, some studies showed that significantly more cases of *S. suis* were observed in a district adjacent to a PRRS district [31, 32]. *S. suis* infection is a zoonotic disease of growing importance in Asia that causes acute meningitis, septicemia and arthritis in humans [33, 34]. More investigations are needed to evaluate the association between the two diseases in Vietnam.

It is well recognized that JE is endemic in Southeast Asia, which is a major cause of viral encephalitis (VE) in young children [35–37]. In Vietnam, national surveillance for VE in humans is ongoing, with the JE virus considered to be a leading cause of VE, accounting for 12–71% of cases [38–40]. JE is a virus transmitted by mosquitoes and pigs are well known as a major amplifying host for transmission to humans [41, 42].

The seroprevalence (63.58%) of JE was similar or slightly lower compared to other studies in Southeast Asia (Laos, Cambodia and Vietnam [65–75%]) but were higher than other Asian countries (Indonesia, Nepal and Taiwan) (73.45%) [8, 43–47]. Bac Giang province was included in both studies, and interestingly seroprevalence had similar levels (73.81% in our study and 79.0% in Lee et al., 2019). Pigs are the most important amplifying hosts for JE virus transmission because they are raised in close proximity to humans [48, 49]. In order to prevent the spread of the disease, it is important to increase awareness amongst pig farmers.

For leptospirosis, our study showed that the seroprevalence was similar to a previous study conducted by Lee et al. (21.05%) [8]. However, it was significantly higher than in another previous study (8.17%) [7]. A possible explanation is that all samples in the previous study were collected from slaughterhouses where healthy or visually good condition pigs were likely to be sent for butchery. Serovar Bratislava and Tarassovi had the highest seroprevalence which were similar to the previous studies [8, 50]. It is known that pigs are the main host for serovar Bratislava, Muenchen, Pomona and Tarassovi [51–53]. Bratislava and Tarassovi in particular have been commonly detected in wild boars [54, 55]. It has been suggested that wild boars may play a role in transmission to domesticated pigs. However, further study is necessary to evaluate this role in wild boars in Vietnam.

In general, various PRRS and PCV vaccines have been introduced for pigs. However, these vaccinations are commonly used in large farms, but not in small farms that are responsible collectively for 70–75% of the total pig production in Vietnam. Because smallholder farmers are able to trade pigs without certification of vaccination, there is no of incentive for smallholders to use these vaccines.

Medium farms are the major suppliers of piglets to smallholders (accounting for more than 70% of pig production), resulting in the hierarchical structure of animal movement from medium to small farms. One the other hand, large farms have better biosecurity and are managed by large commercial companies, are unlikely to have animal movements toward small and medium farms. Our study found that medium farms had relatively higher seroprevalences of diseases compared to small and large farms. Because biosecurity levels of small and small-to-medium farms are low to non-existent, these provide opportunities for the introduction, spill-over and spread of pathogens. Therefore, medium farms need to be targeted to efficiently reduce and prevent the transmission of disease to small farms in Vietnam.

The main limitation in our study was that our samples may not be representative because these were not proportionally collected depending on farm size. We had to take into account the reality that large and medium farmers were not willingly to cooperate (mainly for biosecurity reasons) with our study.

It is well known that farm movements [i.e. direct contact (animal movement) and indirect contact (e.g., vehicles, equipment and personnel) contact] play an important role in between- farm disease transmission [56]. However, few studies have been conducted to demonstrate these patterns in Vietnam. We found that pig famers still have a poor understanding of biosecurity: more than 60% of pig farmers operate a continuous flow system that increase the access of susceptible pigs to objects contaminated by infectious pigs. In addition, it is necessary to improve the accessibility of veterinary services for 40% of pig farmers.

In Vietnam, after the restructuring of government systems in Oct 2017, veterinary services at the district and commune levels were weakened to an extent that early detection/reporting and rapid control/prevention intervention can not be implemented for transboundary animal diseases, emerging and zoonotic diseases. Our study showed that 40% of farms did not receive any veterinary services in the last 6 months.

A typical example is the ongoing outbreak of African swine fever (ASF), which was first detected in February, 2019 at 33-pig farms in Hung Yen, a northern Vietnamese province [57]. By December 2019, more than 5 million pigs have been culled or perished from the disease in all provinces. Poor/slow disease reporting as a result of low compensation rates, uncertainty of time to receive compensation and the complexity of procedures from local authorities was a major reason for the rapid spread of disease across the country. This was further compounded by the generally, low biosecurity levels of small and small-to-medium commercial farms, which provides an opportunity for the introduction of infectious diseases. These are points that would need to be addressed in order to prevent the future spread of infectious diseases in Vietnam.

## Conclusions

Our study provided evidence that PCV2, PRRS and H. hyo are endemic in pigs in Vietnam. Given the economic impacts that these pathogens have elsewhere, the findings confirm the need for studies to evaluate the association between antibody response and clinical relevance as well as to assess the economic impact of co-infections at farm level. We also found that high seroprevalences of JE and leptospirosis were detected in pigs. From a pubic health point of view, it is crucial to raise public awareness for high risk occupations (mainly pig farm workers) who have higher chances to come in contact with infected pigs, contaminated materials and vectors.

## Methods

### Study locations and design

**Fig. 4:**
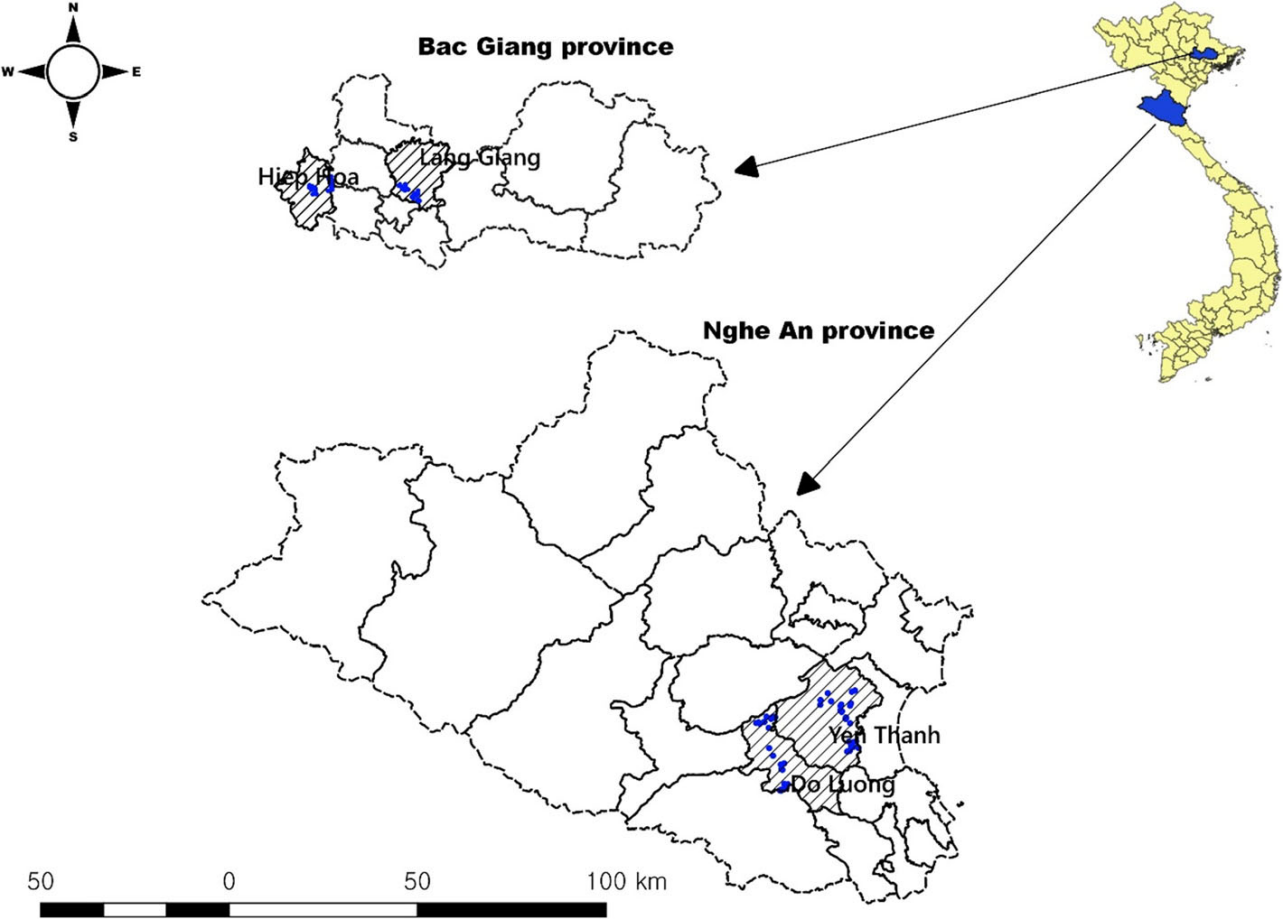
Selected pig farm locations (blue dots) in two districts from two provinces (This figure was created by our own team)

Bac Giang and Nghe An are located in the Red river delta and north central coast regions of Vietnam, with an estimated human population of 1.7 and 3.1 million, respectively (Fig. 4) [58]. As of 2017, there were about 1.08 pigs in Bac Giang and 0.89 million pigs in Nghe An. A total of 120 pig farms (60 farms /province) were randomly selected among registered farms from two provinces. Within each province, two districts were selected. Face-to-face interviews were carried out with adults (> 18 years old) who were mostly involved in pig-rearing. The questionnaires covered demographic information, farm management, vaccination history and farm contact information (on and off farm movements; Additional file 1).

For blood sampling, five pigs (16–20 weeks old) were randomly selected in each farm while farmers were being interviewed. A total of 600 blood samples were collected for evaluating the sero-prevalences of five pathogens [PCV2, PRRS, M. hyo, JE and leptospirosis].

### Laboratory analysis

The sera were extracted after centrifugation and kept at − 20 °C in a cool box until delivery to the National Institute of Veterinary Research (NIVR) in Hanoi. For four pig pathogens (PCV2, PRRS, M. hyo and JE), enzyme-linked immunosorbent assay (ELISA) was used to measure antibodies in pig samples. We followed the guidelines of manufacturer (VDPro PCV2, PRRS, M. hyo and JE AB ELISA; MEDIAN Diagnostics, Chuncheon-si, Gangwon-do, Korea). For leptospirosis diagnosis, the MAT was used to identify the positive samples. The MAT results were recorded by using 2-fold serial dilutions of serum samples, beginning from 1:100 to 1:1600 (end-point). A total of 16 serovars (Table 5) were tested as the highest dilution point that agglutinated > 50% of live leptospires compared to the control samples were recorded. Positivity was considered as MAT titers ≥1:100 for at least one of the tested serovars.

**Table 5:** List of *Leptospira* antigens used in the MAT

No.	Genomospecies	Serogroup	Serovar
1	*L. interrogans*	Australis	Australis
2	L. interrogans	Autumnalis	Autumnaliss
3	L. interrogans	Bataviae	Bataviae
4	L. interrogans	Australis	Bratislava
5	L. interrogans	Canicola	Canicola
6	L. kirschneri	Grippotyphosa	Grippotyphosa
7	L. interrogans	Hebdomadis	Hebdomadis
8	L. interrogans	Icterohaemorrhagiae	Icterohaemorrhagiae
9	L. borgpetersenii	Javanica	Javanica
10	L. noguchii	Panama	Panama
11	L. interrogans	Pomona	Pomona
12	L. interrogans	Pyrogenes	Pyrogenes
13	L. borgpetersenii	Sejroe	Hardjo
14	L. borgpetersenii	Sejroe	Saxkoebing
15	*L. biflexa*	Semaranga	Patoc
16	L. borgpetersenii	Tarassovi	Tarassovi

Source: http://leptospira.amc.nl/leptospira-library/leptospira-strains/?grid-page=2

### Data analysis

The selected farms were classified into types based on number of pigs held by farms: small< 100; medium between 100 and 500; large farms≥500 pigs. The seroprevalence was calculated based on the proportion of positive samples with a 95% Clopper-Pearson/Exact CI. It was calculated by vaccinated and unvaccinated at animal/herd level, respectively as vaccination history was collected. Herd prevalence was estimated by the farm type (small, medium and large), which was defined as positive when at least one sampled pig showed positivity.

For positive pathogens, we assessed the proportion of co-infection with other pathogens. In addition, we evaluated the proportion of accumulative co-infection trends among five pathogens. All data were entered into Microsoft Excel 2016 and analyzed using STATA (version 15.1 StataCorp, College Station, TX, USA). 3.5.2). QGIS (Quantum GIS development Team 2018. QGIS version number 3.6.0) was used to create the map.

## Supplementary information

**Additional file 1.** Summary of survey data for pig farms in Bac Giang and Nghe An province of Vietnam.

**Additional file 2.** Questionnaire for pig farmers.

## Data Availability

Our questionnaires are available (Additional file 2). All datasets supporting our findings are available from the corresponding author on reasonable request.

## Abbreviations

ASF: African swine fever
CI: Confidence interval
CSF: Classical swine fever
ELISA: Enzyme-linked immunosorbent assay
FMD: Foot-and-mouth
HP: highly pathogenic
JE: Japanese encephalitis
M. hyo: Mycoplasma hyopneumoniae
MAT: microscopic agglutination test
NIVR: National Institute of Veterinary Research
PCV2: Porcine circovirus type 2
PRDC: Porcine respiratory disease complex
PRRS: Porcine reproductive and respiratory syndrome
VE: Viral encephalitis
