# Supplementary material for: Seroprevalences of multi-pathogen and description of farm movement in pigs in two provinces in Vietnam
Source: BMC Vet Res. 2020 Jan 14;16:15. doi: 10.1186/s12917-020-2236-7 (PMC6958752; doi:10.1186/s12917-020-2236-7)
Supplement: Supplementary file 1 — Additional file 1. Summary of survey data for pig farms in Bac Giang and Nghe An province of Vietnam. [file 12917_2020_2236_MOESM1_ESM.docx]

Supplementary 1. Summary of survey data for pig farms in Bac Giang and Nghe An province of Vietnam

| Parameters | Value |
| --- | --- |
| Total farms (n) | 120 |
| Small | 89 (74.17%) |
| Medium | 26 (21.67%) |
| Large | 5 (4.17%) |
| Gender (n) |  |
| Female | 26 (26.67%) |
| Male | 94 (73.33%) |
| Education (n) |  |
| None | 3 (2.50%) |
| Primary & middle school | 64 (53.33%) |
| High school | 43 (35.83%) |
| College/university or more | 10 (8.33%) |
| Age (year) |  |
| <30 | 2 (3.33%) |
| 30-39 | 8 (6.67%) |
| 40-49 | 50 (41.67%) |
| 50-59 | 48 (40.0%) |
| ≥60 | 12 (10.0%) |
|  |  |
