# Supplementary material for: Seroprevalences of multi-pathogen and description of farm movement in pigs in two provinces in Vietnam
Source: BMC Vet Res. 2020 Jan 14;16:15. doi: 10.1186/s12917-020-2236-7 (PMC6958752; doi:10.1186/s12917-020-2236-7)
Supplement: Supplementary file 2 — Additional file 2. Questionnaire for pig farmers. [file 12917_2020_2236_MOESM2_ESM.doc]

Questionnaire for pig farmers

Date of interview: _____/______/______ Questionnaire No.: _________

Interviewer: ____________ Farm ID: ______________

**General information**

| 1. Name of interviewee: | Telephone: |
| --- | --- |
| 2. Gender: 🞏1=Male 2=Female | 3. Age (in years): |
| 3. Address of farm |  |
| 4. Education level | 1 = None 4 = High school complete  2 = Primary complete 5 = College or University  3 = Secondary complete |
| 5. Coordinates (GPS) and altitude | Latitude(N/S):______________Longitude(E/W):______________  Altitude: ___________m |

**Pig farm information**

1. Breed of pigs: _________________ (1=exotic, 2=crossbreed, 3=local)

2. Please describe your farms in the table as below

| Pig type | Number |
| --- | --- |
| Piglet (0-4 weeks) |  |
| Weaner (5-8 weeks) |  |
| Grower (9-16 weeks) |  |
| Fattening (> 16 weeks) |  |
| Pregnancy |  |
| Sows |  |
| Boars |  |
| Total number of pigs |  |

3. What types of pig husbandry are you practicing? ___________

1=Free ranging 2=Confinement 3=Other (specify)____________

4. If confinement, total number of pens: ____________

5. Average number of pig per pen: __________

6. Average Size of pen: __________x_________ m

7. Type of pen: ___________ (1=ground level, 2=elevated, 3=other)

8. Type of enclosure: ___________ [1= cement), 2=Post-and-rail fence, 3=other]

9. Type of roofing: __________ (1=no roof, 2=Thatched, 3=tiles, 4=iron sheet, 5=other)

10. Type of flooring: ___________(1=soil or sand, 2=cement, 3=wood, 4=other)

11. Type of housing for sows and gilts: ________ (1=individual stall, 2=group housing. 3=Other

12. Presence of other pig farm(s):____________(1<100m, 2<500m, 3≥ 500m)

13. Do you have any other animals/livestock on your farm? (Yes 🞏 No 🞏)

If yes, please specify ___________________

14.How often do you disseminate your semen to other farms?

1= Once a week 2= every two weeks 3= once a month 4= every three months = other _________

15. Have you ever seen any wildlife (including wild pigs) near your farms over the last 6 months?

(Yes 🞏 No 🞏) If yes, specify________________

**Health status of the farms**

1. Do you seek external assistance when your pigs are sick? (Yes 🞏 No 🞏)

If yes, from who 1=Veterinarian/Paravet 2=Governmental staff 3=Another farmers

4=Others (Specify: ___________________)

2. For the last two months how many times was your farm visited for any purpose by

1=Veterinarian/Paravet: _________ 2=Governmental staff:_________ 3=Another farmers: _________

4=Others (Specify: _______________)

**3. Please describe the number of main clinical signs and *morbidity/mortality* rates in your pigs over the last two months.**

| **Pig type** | **Piglet**  **(0-4wks)** | **Weaner**  **(5-8 wks)** | **Grower**  **(9-16 wks)** | **Fattening**  **(> 16 wks)** | **Pregnancy** | **Sows** | **Boars** |
| --- | --- | --- | --- | --- | --- | --- | --- |
|  | **Sick & death no./total** | **Sick & death no./total** | **Sick & death no./total** | **Sick & death no./total** | **Sick & death no./total** | **Sick & death no./total** | **Sick & death no./total** |
| Nerve system disease |  |  |  |  |  |  |  |
| [Gastrointestinal disease](http://endic.naver.com/enkrIdiom.nhn?idiomId=abc9b2465ef94aaf9588dda65cf1a871&query=소화기계통) |  |  |  |  |  |  |  |
| Respiratory  Disease |  |  |  |  |  |  |  |
| Reproductive disease |  |  |  |  |  |  |  |
| Other (specify_________) |  |  |  |  |  |  |  |
| Total |  |  |  |  |  |  |  |

# 4. Please describe the major outbreaks of diseases in your pigs over the two months

| **Pig diseases** | **Outbreak**  **of disease** | Please describe the number of sick and death pigs in your pigs | | | |
| --- | --- | --- | --- | --- | --- |
|  |  | **Current month**  **(sick and death no./total)** | | **one month ago**  **(sick no. and death /total)** | |
| **FMD** | Yes 🞏 No 🞏 |  |  |  |  |
| **CSF** | Yes 🞏 No 🞏 |  |  |  |  |
| **PRRS** | Yes 🞏 No 🞏 |  |  |  |  |
| **PCV2** | Yes 🞏 No 🞏 |  |  |  |  |
| **PED** | Yes 🞏 No 🞏 |  |  |  |  |
| **Other diseases**  **_______** | Yes 🞏 No 🞏 |  |  |  |  |

**Animal contact information**

- *Pig direct contact*

1. Where did you import/ source your pigs from over the last 6 months? _________

1= Farmer from the same village 2=Farmer from another village 3= another commune 4= another province 5=other (specify_____________)

*If yes, please describe the name of village(s) or market(s) or district/commune/province(s) and size of farm (estimated number of pigs).* _________________________

2. How often did you import the pigs on your farm over the last 6 months? _____________

*If yes, please describe the number/age of pigs that you did import? _____________*

3. What kind of production system use on your farm? ___________

1=All-in-All-out *(keeping animals together in groups. Animals from different groups are not mixed during their stay on the farm)*

2=Continuous flow *(pigs move as individuals, not as closely matched age groups, and a facility is never totally emptied because pigs or sows are always moving through it)*

4. Did any breeding male arrive on your farms over the last 6 months? (Yes 🞏 No 🞏)

*If yes, how often _______________*

5. Were the newly arriving breeding males always, sometimes, or never put though an isolation or quarantine process when being introduced to the site?

1=Always 2=Sometimes 3=Never If Always or Sometimes, how many days did it last? ____________

6. Where did you export your pigs over the last 6 months? ___________

1= Farmer from the same village 2=Farmer from another village 3= another commune 4= another province 5=other (specify_____________)

*If yes, please describe the name of village or market or district/commune/province(s) and size of farm (estimated number of pigs). ___________________________*

7. How often did you export your pigs from your farm over the last 6 months? ___________

8. Have your pigs (any pigs) ever been in contact with neighbouring pigs (Yes 🞏 No 🞏)

If yes, how far is the close neighbouring farm ____________m

- *Transportation and equipment contacts*

9. How do you transport your pigs between farms/villages and markets?

1=Vehicle 2=Motorbike 3=Bicycle 4=Other (specify: ____________)

10. How often your pigs can be transported (import and export) on your farm over the last 6 months? _______________

11. Do you share any equipment with other farms? (Yes 🞏 No 🞏)

If yes, please describe the list of items ___________________

12. if yes (question 11), how often do you share it over the last 6 months?

_______________

- *Personal contacts*

13. How often do other farmers visit your farm over the last 6 months? _____________

14. How often do veterinarians or animal health workers visit your farm over the last 6 months? ____________

15. How often do traders visit your farm over the last 6 months? _______________

16. How often do strangers (except 13-15) your farm over the last 6 months? ___________

*If yes, please describe the list of strangers* ____________________________

**Assessment of biosecurity**

*Workers and visitors*

1. Do workers and visitors enter your farm without restriction Yes 🞏 No 🞏

2. If no, do workers/visitors wear protective clothing or footwear? Yes 🞏 No 🞏 Other (specify______________)

*Vehicles and equipment*

1. Do drivers or vehicles enter your farm without restriction? Yes 🞏 No 🞏

mixed. If no, what do you do? ___________________

2. Do you regularly clean/disinfect all instruments on your farm (such as restraint snares, needles and scalpels)?

Yes 🞏 No 🞏

3. Do you share equipment between farms? Yes 🞏 No 🞏

*Control of pests*

1. Do you regular pest and rodent control, with rodenticides? Yes 🞏 No 🞏

2. Do you regular clean/remove the residues of pig feed? Yes 🞏 No 🞏

*Introduction of new pigs*

1. Do you isolate the newly purchased pigs in a quarantine pen? Yes 🞏 No 🞏

2. If yes, how long? _________________

3. In addition, during the quarantine, do you observe the new pigs whether they are sick or not? Yes 🞏 No 🞏

4. Do you do vaccination and deworming for the new pig(s) ? Yes 🞏 No 🞏

5. Do you use artificial insemination (AI) on your farm?

*Age Segregation*

1. Do you segregate the pigs by age group (born in the same week and in the same room)?

Yes 🞏 No 🞏

2. Do you use an all-in-all-out hygiene policy? Yes 🞏 No 🞏

3. If yes, do you clean/disinfect the premises? Yes 🞏 No 🞏

*Cleaning and disinfection*

1. Do you regularly clean on your farm Yes 🞏 No 🞏

2. If yes, do you use any detergent or disinfectant? Yes 🞏 No 🞏

3. Do you remove manure/urine? Yes 🞏 No 🞏 If yes, how often? ________________

4. Do you remove straw bedding where sick and dead pigs have been present? Yes 🞏 No 🞏

*Other pre-emptive measures*

1. Do you use any of these identifiers (such as ear tag, tattoo and mark pig)? Yes 🞏 No 🞏

2. Do you have periodic herd health inspections (audits) from veterinarians and animal health workers? Yes 🞏 No 🞏 . If yes, how often? ________________

3. If you find any major events related to disease occurrence, do you contact a veterinarians or health animal workers? Yes 🞏 No 🞏 6. If no, how do you do? ______________________

4. How to dispose of a dead pig(s) (select more than two)?

1=Throw away (Yes 🞏 No 🞏) 2=Bury (Yes 🞏 No 🞏) 3=Burn (Yes 🞏 No 🞏) 4=Eat (Yes 🞏 No 🞏)

5=Sell (Yes 🞏 No 🞏) 6=Other (specify__________________)
